# Supplementary material for: Associations of Diet Quality and Heavy Metals with Obesity in Adults: A Cross-Sectional Study from National Health and Nutrition Examination Survey (NHANES)
Source: Nutrients. 2022 Sep 28;14(19):4038. doi: 10.3390/nu14194038 (PMC9571327; doi:10.3390/nu14194038)
Supplement: Supplementary file 1 [file nutrients-14-04038-s001.zip › nutrients-1891605-supplementary.pdf]

Supplementary Materials

Table S1. Healthy Eating Index-2015 components, point values, and standards for scoring

Table S2. Sensitivity analysis by excluding participants with diabetes and cardiovascular disease for the association of HEI-2015 total scores and heavy metals with obesity in NHANES 2007-2018 (N=13,049).

Table S3. Sensitivity analysis for the association between HEI-2015 total scores and heavy metals with obesity using NHANES data during 2003-2018 (N=25,155).

Table S1. Healthy Eating Index-2015 components, point values, and standards for scoring.

Component	Maximum Points	Standard for Maximum Score	Standard for Minimum Score of Zero
**Adequacy:**			
Total Fruits [b]	5	≥0.8 cup equiv. per 1,000 kcal	No Fruit
Whole Fruits [c]	5	≥0.4 cup equiv. per 1,000 kcal	No Whole Fruit
Total Vegetables [d]	5	≥1.1 cup equiv. per 1,000 kcal	No Vegetables
Greens and Beans [e]	5	≥0.2 cup equiv. per 1,000 kcal	No Dark Green Vegetables or Legumes
Whole Grains	10	≥1.5 oz equiv. per 1,000 kcal	No Whole Grains
Dairy [e]	10	≥1.3 cup equiv. per 1,000 kcal	No Dairy
Total Protein Foods [f]	5	≥2.5 oz equiv. per 1,000 kcal	No Protein Foods
Seafood and Plant Proteins [f, g]	5	≥0.8 oz equiv. per 1,000 kcal	No Seafood or Plant Proteins
Fatty Acids [h]	10	(PUFAs + MUFAs)/SFAs ≥2.5	(PUFAs + MUFAs)/SFAs ≤1.2
**Moderation:**			
Refined Grains	10	≤1.8 oz equiv. per 1,000 kcal	≥4.3 oz equiv. per 1,000 kcal
Sodium	10	≤1.1 gram per 1,000 kcal	≥2.0 grams per 1,000 kcal
Added Sugars	10	≤6.5% of energy	≥26% of energy
Saturated Fats	10	≤8% of energy	≥16% of energy

Note:

[a] Intakes between the minimum and maximum standards were scored proportionately.

[b] Included 100% fruit juice.

[c] Included all forms except juice.

[d] Included legumes (beans and peas).

[e] Included all milk products, such as fluid milk, yogurt, and cheese, and fortified soy beverages.

[f] Included legumes (beans and peas).

[g] Included seafood, nuts, seeds, soy products (other than beverages), and legumes (beans and peas).

[h] Ratio of poly- and monounsaturated fatty acids (PUFAs and MUFAs) to saturated fatty acids (SFAs).

Table S2. Sensitivity analysis by excluding participants with diabetes and cardiovascular disease for the association of HEI-2015 total scores and heavy metals with obesity in NHANES 2007-2018 (N=13,049).

Exposure	N	Peripheral Obesity [a] [OR (95%CI)]	Abdominal Obesity [b] [OR (95%CI)]
HEI-2015 total score			
Quartile 1[c] (≤43.7)	3,247	1.00 (Ref)	1.00 (Ref)
Quartile 2 (>43.7 and ≤53.3)	3,281	0.82 (0.70, 0.96)	0.88(0.75, 1.03)
Quartile 3 (>53.3 and ≤63.4)	3,313	0.68 (0.58, 0.80)	0.67 (0.56, 0.79)
Quartile 4 (>63.4)	3,208	0.46 (0.39, 0.54)	0.51 (0.45, 0.59)
P for trend [d]	—	<0.001	<0.001
Continuous (per IQR)	13,049	0.65 (0.60, 0.71)	0.67(0.62, 0.73)
Pb			
Quartile 1[c] (≤0.65)	2,945	1.00 (Ref)	1.00 (Ref)
Quartile 2 (>0.65 and ≤1.05)	3,121	0.76 (0.65, 0.89)	0.79 (0.66, 0.94)
Quartile 3 (>1.02 and ≤1.60)	3,263	0.62 (0.52, 0.73)	0.77 (0.65, 0.91)
Quartile 4 (>1.60)	3,720	0.43 (0.35, 0.52)	0.50 (0.41, 0.60)
P for trend [d]	—	<0.001	<0.001
Continuous (per IQR)	13,049	0.90 (0.82, 0.98)	0.90 (0.85, 0.95)
Cd			
Quartile 1[c] (≤0.17)	2,925	1.00 (Ref)	1.00 (Ref)
Quartile 2 (>0.17 and ≤0.28)	3,055	0.86 (0.74, 1.00)	0.88 (0.76, 1.00)
Quartile 3 (>0.28 and ≤0.50)	3,389	0.67(0.57, 0.78)	0.70 (0.59, 0.82)
Quartile 4 (>0.50)	3,680	0.47 (0.39, 0.58)	0.57 (0.48, 0.68)
P for trend [d]	—	<0.001	<0.001
Continuous (per IQR)	13,049	0.92 (0.87, 0.97)	0.94 (0.91, 0.99)
Hg			

Quartile 1[c] (≤0.45)	3,279	1.00 (Ref)	1.00 (Ref)
Quartile 2 (>0.45 and ≤0.87)	3,333	0.89 (0.78, 1.02)	0.99 (0.85, 1.15)
Quartile 3 (>0.87 and ≤1.78)	3,233	0.84 (0.71, 0.98)	0.81 (0.68, 0.96)
Quartile 4 (>1.78)	3,204	0.55 (0.46, 0.65)	0.56 (0.48, 0.66)
P for trend [d]	—	<0.001	<0.001
Continuous (per IQR)	13,049	0.85 (0.81, 0.89)	0.86 (0.83, 0.90)

Note: CI, confidence interval; OR, odds ratio; IQR, interquartile range; Pb, Lead; Cd, Cadmium; Hg, Total mercury. All results were adjusted for age, sex, race, education level, marital status, income, smoking, alcohol consumption, and physical activity.

[a] Peripheral obesity was defined as BMI ≥ 30 kg/m^2.

[b] Abdominal obesity was defined as a WC of ≥102 cm for males and ≥ 88 cm for females.

[c] Least HEI-2015 total score quartile.

[d] *P* values for trend were derived based on ordinal quartile values.

Table S3. Sensitivity analysis for the association of HEI-2015 total scores and heavy metals with obesity using NHANES data during 2003-2018 (N=25,155)

Exposure	N	Peripheral Obesity [a] [OR (95%CI)]	Abdominal Obesity [b] [OR (95%CI)]
HEI-2015 total score			
Quartile 1[c] (≤43.4)	6,177	1.00 (Ref)	1.00 (Ref)
Quartile 2 (>43.4 and ≤52.7)	6,355	0.82 (0.73, 0.91)	0.87(0.78, 0.97)
Quartile 3 (>52.7 and ≤62.5)	6,373	0.68 (0.61, 0.76)	0.70 (0.63, 0.79)
Quartile 4 (>62.5)	6,250	0.47 (0.42, 0.52)	0.51 (0.45, 0.57)
P for trend [d]	—	<0.001	<0.001
Continuous (per IQR)	25,155	0.66 (0.62, 0.70)	0.68 (0.64, 0.73)
Pb			
Quartile 1[c] (≤0.71)	5,548	1.00 (Ref)	1.00 (Ref)
Quartile 2 (>0.71 and ≤1.11)	5,914	0.87 (0.76, 0.96)	0.86 (0.76, 0.98)
Quartile 3 (>1.11 and ≤1.74)	6,291	0.65(0.58, 0.73)	0.75 (0.66, 0.85)
Quartile 4 (>1.74)	7,402	0.49 (0.43, 0.56)	0.56 (0.49, 0.65)
P for trend [d]	—	<0.001	<0.001
Continuous (per IQR)	25,155	0.87 (0.82, 0.93)	0.89 (0.85, 0.93)
Cd			
Quartile 1[c] (≤0.18)	5,367	1.00 (Ref)	1.00 (Ref)
Quartile 2 (>0.18 and ≤0.30)	6,232	0.84 (0.75, 0.95)	0.83 (0.73, 0.94)
Quartile 3 (>0.30 and ≤0.55)	6,717	0.72 (0.63, 0.82)	0.73 (0.64, 0.83)
Quartile 4 (>0.55)	6,839	0.50 (0.43, 0.58)	0.52(0.45, 0.59)
P for trend [d]	—	<0.001	<0.001
Continuous (per IQR)	25,155	0.91 (0.88, 0.95)	0.93 (0.90, 0.96)
Hg			
Quartile 1[c] (≤0.45)	6,428	1.00 (Ref)	1.00 (Ref)

Quartile 2 (>0.45 and ≤0.87)	6,478	0.97 (0.87, 1.07)	0.97 (0.86, 1.10)
Quartile 3 (>0.87 and ≤1.74)	6,208	0.91 (0.80, 1.04)	0.93 (0.72, 0.95)
Quartile 4 (>1.74)	6,041	0.62 (0.55, 0.71)	0.60 (0.53, 0.68)
P for trend [d]	—	<0.001	<0.001
Continuous (per IQR)	25,155	0.87 (0.84, 0.89)	0.88 (0.85, 0.91)

Note: CI, confidence interval; OR, odds ratio; IQR, interquartile range; Pb, Lead; Cd, Cadmium; Hg, Total mercury. All results were adjusted for age, sex, race, education level, marital status, income, smoking, alcohol consumption, diabetes and cardiovascular disease.

[a] Peripheral obesity was defined as BMI ≥ 30 kg/m^2.

[b] Abdominal obesity was defined as a WC of ≥102 cm for males and ≥ 88 cm for females.

[c] Least HEI-2015 total score quartile.

[d] *P* values for trend were derived based on ordinal quartile values.
